# Cord care practices and related outcomes among caregivers in two referral facilities in Ghana: a cross-sectional study

**DOI:** 10.21203/rs.3.rs-6401569/v1

**Published:** 2025-05-12

**Authors:** Gloria Amponsah-Kodua, Peter Agyei-Baffour, Paul Okyere, Princess Ruhama Acheampong, Julius Kwabena Karikari, Kofi Akohene-Mensah, Nadia Tagoe, Justine Naab Ti-Baliania, Ellis Owusu-Dabo

**Affiliations:** Kwame Nkrumah University of Science and Technology; Kwame Nkrumah University of Science and Technology; Kwame Nkrumah University of Science and Technology; Kwame Nkrumah University of Science and Technology; University Hospital, Kwame Nkrumah University of Science and Technology; Kwame Nkrumah University of Science and Technology; Kwame Nkrumah University of Science and Technology; Kwame Nkrumah University of Science and Technology; Kwame Nkrumah University of Science and Technology

**Keywords:** Cord care, Chlorhexidine, Methylated spirit, Cord bleeding, Cord granuloma, Cord infection, Related outcome

## Abstract

**Background:**

Neonatal mortality is still high in Ghana and Sub-Saharan Africa though great strides have been made in other parts of the world. Neonatal infection causes a third of neonatal deaths. The umbilical stump can be an entry point for bacteria if not properly cared for, leading to omphalitis and sepsis. The World Health Organisation and Ghana Health Service recommend using 7.1% chlorhexidine digluconate for cord care to reduce the incidence of cord complications. There is however inadequate data on its usage and cord outcomes compared to other cord care methods. This study aimed to assess the risk of cord complications with the various cord care practices in two referral facilities in the Ashanti region, Ghana.

**Methods:**

A cross-sectional study with a quantitative approach was conducted from June to December 2023. Simple random sampling was used to select 453 caregivers. We collected data on cord care practices and outcomes using a questionnaire. Stata/SE Version 17.0 was used to analyse the data.

**Results:**

Antenatal clinic attendance significantly reduced the odds of cord infection (aOR = 0.03, p-value = 0.018). Babies of caregivers who washed their hands before cord care were at a decreased odds of getting cord infection (aOR = 0.20, p-value = 0.047). Babies were at increased odds (aOR = 42, p-value = 0.010) of cord bleeding if their caregivers received recommendation on cord care from people other than health workers. There was no statistically significant difference in cord complications (i.e. cord bleeding, cord granuloma and cord infection) in the chlorhexidine and the methylated spirit group (p-value > 0.05). Recall bias was a limitation of the study since caregivers of children between one week and one year were required to report cord practices and outcomes in the first few weeks of their babies’ lives.

**Conclusions:**

The cord outcome differs with the various cord care practices. Antenatal clinic attendance should be encouraged and education on proper cord care practices should be intensified among caregivers. Randomized control trials or cohort studies should be done to compare the cord outcome in chlorhexidine and methylated spirit.

## Introduction

Neonatal cord care practices are important in the newborn period. Preventing cord infection can reduce neonatal mortality, which is high in Sub-Saharan Africa (SSA). Cord care is the series of steps applied in handling the umbilical cord after the delivery of the newborn [1]. The umbilical cord connects the foetus in the womb to the mother and is cut immediately after delivery. The stump typically takes one to three weeks to fall off and heal.

According to the World Health Organization (WHO), SSA has the highest neonatal mortality rate (NMR) in the world (27 deaths per 1000 live births) [2]. Neonatal infections account for a third of all neonatal deaths [3] and 75 percent of newborn infection-related deaths occur in the first week of life with the umbilical cord serving as the entry point for these infections [4]. Blumenroder et al., [5] in Western Tanzania showed in their study, a prevalence of 22% for neonatal infections. The prevalence of cord infection in Ghana is unknown, however, a study done in a teaching hospital in the Volta region of Ghana suggested that there was a significant burden of sepsis among neonates and young infants [6]. The technique used for cord care affects how quickly the cord separates, how much the cord bleeds, how well the caregivers comply with instructions and caregiver satisfaction [7].

In 2014, WHO recommended the use of 7.1% chlorhexidine digluconate aqueous solution or gel in neonates born in hospitals in countries with high neonatal mortality (NMR > 30/1000live births) to replace the use of harmful substances such as cow dung. This was on the background that chlorhexidine application to the umbilical cord stump reduced neonatal mortality in countries with high NMR. Though studies done in Tanzania and Zambia; countries with low NMR did not show any statistically significant reduction in NMR [8, 9], trials done in countries with high NMR in Southeast Asia showed substantial decreases in NMR where chlorhexidine was used for cord care [10]. Another study in Bangladesh (community-based), showed that applying chlorhexidine to the cord after birth reduced colonisation of the stump with bacteria and reduced severe cord infection and neonatal sepsis in developing countries [11]. Ghana adopted this recommendation because 31% of newborn deaths were from infections [12]. It was to replace the use of harmful substances and even methylated spirit for cord care to help prevent cord infections and reduce neonatal mortality [11]. Since the adoption of chlorhexidine use in Ghana, there haven’t been any studies to compare its outcome with the other unapproved cord care practices still being used [12]. The study therefore sought to ascertain the association between a particular cord care method and the risk of negative cord outcome.

## Methods

### Study design and population

We conducted a facility-based cross-sectional study among caregivers of children aged between 1 week and 1 year who visited the two hospitals during the study period. We excluded caregivers with babies who had undergone umbilical catheterization or other procedures on their cord from the study. We also excluded caregivers who were absent when the child was a neonate.

### Study setting

The Ejisu government hospital is in the Ashanti region of Ghana. It provides health services for people in the municipality and serves as a referral centre for sick children in the neighbouring districts. The Kumasi South Hospital serves as the Ashanti Regional Hospital and receives referrals from hospitals in the region. Both facilities have a robust neonatal unit. The study was conducted from June to December 2023.

### Sample size determination and sampling techniques

The sample size was calculated using the Cochrane formula, n=z^2^pq/d^2^, with a 95% confidence interval, Z = 1.96. We assumed that the proportion of the outcome of interest was 50% since the prevalence of cord care practices was unknown. Therefore p = 0.5 and q, which is 1-p = 0.5. We used a desired precision of 0.05. This gave a sample size of 384. Allowing for 10% possible losses, the total sample size was 422. However, we ended up getting 453 respondents, and we included all of them in the analysis. A simple random sampling method was used to select the study participants.

### Data collection techniques and data quality control

We developed a questionnaire after an extensive literature review and pretested it. The questionnaire contained five sections, which was then entered into Kobo Collect for data collection (Supplementary File 1). The interviewers, who were research assistants, then administered this structured questionnaire under the supervision of the principal investigator. There were three research assistants. One was a health information officer who worked at the Ashanti Regional Hospital, one of the study sites. The second was an MPhil student with a degree in biochemistry, and the third was a teaching assistant in the Department of Biochemistry at KNUST. All of them had prior training in the use of Kobo Collect. The Principal Investigator, a Paediatrician trained them on the aims of the research and the questionnaire administration.

### Data processing and analysis

Data were extracted into an Excel spreadsheet and subsequently cleaned, coded, and analyzed using Stata/SE Version 17.0. Continuous variables were evaluated for normality using the Shapiro-Wilk test and visualized through Q-Q plots and histograms. Normally distributed variables were presented as means with standard deviations, while skewed variables were reported as medians with interquartile ranges (IQR). Categorical variables were summarized as frequencies and percentages. Associations between demographic characteristics, cord care practices, and the three adverse cord care outcomes (cord bleeding, cord granuloma, and cord infection) were assessed using Pearson’s chi-squared test. Fisher’s exact test was applied for categorical variables with expected cell frequencies less than five to ensure statistical validity. For group comparisons, T-tests were used for normally distributed continuous variables, and the Wilcoxon rank-sum test was employed for non-parametric continuous data.

All the independent variables were included in bivariate logistic regression models. Independent variables with p-values < 0.05 in the bivariate logistic regression were then included into a backward stepwise multivariable logistic regression analysis to identify demographic and cord care practices associated with the three adverse cord care outcomes. Variables with p-values < 0.05 in the multivariable model were considered statistically significant predictors of adverse cord care outcomes.

### Ethical considerations

The study received ethical approval from the Committee on Human Research, Publications, and Ethics (CHRPE) of the Kwame Nkrumah University of Science and Technology (KNUST), Ghana. Official letters were submitted to the administrative bodies of the two hospitals involved, and permission was obtained to conduct the research at these facilities. Detailed information about the study was explained to the caregivers, and their consent was sought by signing the Informed Consent Form. Participants were assured of the confidentiality of the data provided and the anonymity of their identity.

## Results

### Sociodemographic data of study participants

Among the 453 participants included in the study, most were recruited from Ejisu Government Hospital (61.6%). The median age of the children was 94 days (IQR: 60–180), and the mean maternal age was 29.8 years (± 5.3). Regarding maternal education, the largest proportion of mothers had completed junior high school (36.9%). Most mothers were employed (80.1%), married (69.1%), and identified as Christian (94.7%). Nearly all mothers (99.6%) attended antenatal care, and the median monthly income was 500 GHC (IQR: 200–750) (Table 1).

### Demographic characteristics and cord care practices with cord bleeding experience

There was a significant difference in monthly income between mothers whose infants experienced cord bleeding and those whose infants did not, with mothers of infants without cord bleeding reporting a higher median income (500 GHC vs. 0 GHC, p-value = 0.027). Additionally, cord exposure after drying was significantly associated with cord bleeding, with a lower proportion of infants who had their cords exposed experiencing bleeding compared to those whose cords were not exposed (1.9% vs. 5.3%, p-value = 0.049) (Table 1).

### Demographic characteristics and cord care practices with cord granuloma experience

There were no statistically significant differences between infants with and without cord granuloma based on the demographic characteristics and cord care practices analyzed (p-values > 0.05) (Table 2).

### Demographic characteristics and cord care practices with cord infection experience

There were no statistically significant differences between infants with and without cord infection based on the demographic characteristics and cord care practices analyzed (p-values > 0.05) (Table 3).

### Association between significant predictors with cord bleeding outcome

After adjusting for potential confounders, recommendations from others (besides health staff or relatives) were significantly associated with an increased odds of cord bleeding compared to health staff recommendations (aOR = 42.00, 95% CI: 2.45– 720.21, p-value = 0.010). Washing hands before cord cleaning was significantly protective against cord bleeding, reducing the odds by 87% compared to those who did not wash their hands after adjusting for potential confounders (aOR = 0.13, 95% CI: 0.03–0.67, p-value = 0.015) (Table 4).

### Association between significant predictors with cord granuloma outcome

No significant predictors of cord granuloma were identified in the logistic regression analysis after adjusting for potential confounders (Table 5).

### Association between significant predictors with cord infection outcome

After adjusting for potential confounders, mothers who attended antenatal care had significantly reduced odds of cord infection compared to those who did not (aOR = 0.03, 95% CI: 0.00–0.56, p = 0.018). After adjusting for other potential confounders, washing hands before cleaning the cord was also significantly protective against cord infection (aOR = 0.20, 95% CI: 0.04–0.98, p = 0.047) (Table 6).

## Discussion

In an effort to reduce cord complications in newborns and improve cord outcomes, a variety of cord practices are employed by caregivers. Some of them, however, lead to adverse outcomes. The prevalence of 3 negative outcomes and their relationship with the various practices were analyzed in this study.

### Cord care practices

The popular use of methylated spirit for cord care by caregivers in this study (89.8%) is similar to findings in a study conducted in the Ashanti region of Ghana [13], where the majority (80.2%) of the caregivers used methylated spirit. In that study, only 6.6% of participants used other unapproved substances such as herbs and toothpaste, which also agrees with our study findings where only 6% of the caregivers used such harmful substances.

The similar findings could be explained by the fact that both studies, though conducted in different sites were still in the same geographic region. In another study conducted by Asiedu et. al., [12] in the Volta region of Ghana, only 35.7% of caregivers used methylated spirit. In that study, the majority (64.3%) used unapproved substances such as toothpaste, salt, shea butter, and herbs. In both studies, there was no mention of chlorhexidine use by participants which may have been because of the unavailability of chlorhexidine or lack of knowledge on the current cord care policy. In Uganda however, Turyasiima et. al., [14] discovered that 30% of the participants used chlorhexidine for cord care which was higher than the 3.3% found in our study. The low patronage of chlorhexidine in our study could be as a result of the unavailability of chlorhexidine and the lack of awareness of the current policy among caregivers. Interestingly, a study in Pumwani Maternity Hospital, Kenya [15], revealed that dry cord care was most prevalent (55%), followed by methylated spirit (25%), the use of saliva (10%), and water (10%).

Our study also revealed that a high percentage of caregivers (96.9%) washed their hands with soap and water before cord care. This was much higher than the findings in a study done in Nigeria where hand washing with soap and water was only conducted by 47% of caregivers [1]. The appropriate hand hygiene by the majority of the caregivers in our study may be because almost all (99.6%) of them attended ANC and must have been educated on proper hand hygiene by the hospital staff.

### Prevalence of negative outcomes, and associations

#### Cord bleeding

In this study, a small fraction (2.9%) of babies experienced cord bleeding. The low prevalence of cord bleeding could have also contributed to the low prevalence of cord infections in the study since blood is an effective medium for bacterial growth. Dessalegn et al., [16] also asserted that, if individuals received incorrect or inadequate advice on cord care, there might be an association with adverse outcomes, including bleeding. The majority of the participants attended ANC and had recommendations on the cord care practice from health workers. Their compliance could have resulted in the low prevalence of cord bleeding.

The only variable that showed a significant statistical association with cord bleeding experience was the mother’s income (p-value = 0.027). One possible explanation could be that mothers with lower median income are less likely to have access to recommended agents for appropriate cord care in the form of chlorhexidine or methylated spirit. Some may thus resort to the use of inappropriate methods of cord care which could induce the risk of cord bleeding. This may not be surprising as some previous studies have reported that mothers from low socio-economic background are less likely to adhere to good cord care practices [17, 18].

Further, it is also possible that mothers with low median income may face related challenges pertaining to limited health literacy which could adversely affect their ability to correctly adhere to appropriate cord care practices and handle any complications associated with umbilical cord care including bleeding. Families with lower income may also live in environments with poor sanitation, increasing the risk of cord infections and cord bleeding.

#### Cord granuloma

An umbilical cord granuloma is the most common benign umbilical pathology in newborns [19]. The cord granuloma prevalence (0.4%) in our study was much lower than the findings in a study done at Hamamatsu University Hospital, Japan, which showed an incidence rate of 5.9% in 10 years [20]. In that same study, Gestational age, male sex, birth weight, and incidence of meconium-stained amniotic fluid were significantly associated with umbilical granuloma development. Another study done in Third-line Hospital, Turkey found a prevalence of 3.8% [21]. Bathing the baby (moisture) before umbilical cord separation was associated with granuloma development in that study. Our study however did not reveal a significant association between cord care practice or demographics and cord granuloma formation. According to Das, the aetiology of cord granuloma is not fully known, but the mode of delivery, umbilical care method, moisture in the umbilicus, infection, and delayed cord separation are the factors that are thought to be associated with cord granuloma formation [22]. In our study, the participants had their baby’s cords fall off in the first week (4–7) days. A significant number (70.6%) also exposed the cord after cleaning it, thereby reducing moisture. The proportion of cord infection (3.5%) was also significantly low. These factors, even though our study did not reveal a significant association, could have accounted for the low incidence of granuloma in this study based on existing data.

#### Cord infection

In our study, only a small fraction (6.8%) of the participants had babies with cord infection. It indicated a lower cord infection prevalence. This was similar to the findings of a community-based cluster randomized trial done in Nepal which showed an infection prevalence of 5–6% [23]. Other studies, however, have shown a higher cord infection prevalence. For instance, Turyasiima and his colleagues found a high cord infection prevalence of 27.1% in a hospital-based cross-sectional study done in Uganda [14]. In that study, it was asserted that umbilical cord infection risk is 62% higher in neonates receiving topical cord applications of potentially unclean substances. Unplanned home birth or septic delivery, lack of knowledge of cord care, and prolonged rupture of membranes were also cited as risk factors.

Our study observed that 93.1% of the respondents used substances recognized as appropriate antiseptics (methylated spirit and chlorhexidine) for cord care. Most of them were also delivered in the hospital and not at home where unsterilized tools would have been used for cord cutting. Many of them (99.6%) had attended ANC and complied with the health workers’ recommendations. This could have accounted for the low cord infection.

The substance used for cord care, exposure of the cord after cleaning it, and handwashing practices were not significantly associated with cord infection in this study. This differs from the findings in a study done in Kenya where it was observed that poor cord hygiene (not washing hands with soap and water, applying substances other than chlorhexidine or methylated spirit and not leaving the cord exposed) increased the odds of neonatal sepsis [24]. It was implied that sixty-seven percent of neonatal sepsis cases would have been prevented in the study population if proper cord hygiene had been practiced. In that study, chlorhexidine and methylated spirit application were found to reduce the odds of infection. A study done in Nigeria, also revealed that unhygienic cord practices increased the risk of infection in newborns [25]. Thus, handwashing with soap, preferably under running water, significantly reduces infections [26]. Our study was done in the post-Covid period. During the Covid and immediate post-Covid period, there was a surge in the use of alcohol-based hand sanitizer which is a potent antibacterial with similar effects as hand washing [27]. This could have served as a confounder to the association between handwashing and cord infection.

Even though the level of education was not significantly associated with the development of cord infection in our study, Turyasiima et al. discovered that the level of education was associated with the risk of cord infection in Uganda [14]. Mothers who had only received secondary education were at an increased risk of developing cord infections. In our study, 92.1% of the participants responded that the cord practice was a recommendation from health workers. It can be extrapolated that they complied with other instructions given. Understanding sound advice, and adherence to it could have led to positive effects irrespective of the educational level and could have also served as a confounder to the relationship between education and cord infection.

### Chlorhexidine versus methylated spirit

Though has been a recommendation that chlorhexidine should be used for cord care even for babies born in health centres because it significantly reduces cord infections [28], and a randomized control trial done in Kenya asserted that chlorhexidine significantly reduced the incidence of omphalitis compared to dry cord care and methylated spirit [15], our study generally did not reveal that negative cord outcomes such as cord infections, cord granuloma, and cord bleeding were higher in the group of caregivers who used methylated spirit compared to chlorhexidine. This could be attributed to the fact that methylated spirit is an antiseptic that can also inhibit organisms’ growth if applied properly. In this study, almost all the participants attended ANC. The majority used the cord care method recommended by a health staff, who would most likely teach its correct application. This finding in our research is also supported by studies done in Zambia which showed no significant reduction in NMR with chlorhexidine compared to other regimens [9]. Shwe et al., also discovered in their study that methylated spirit was not inferior to chlorhexidine in the prevention of cord infection [17].

### Approved substances versus unapproved substances

Although there are several studies to support the assertion that unapproved substances such as shea butter, herbs and toothpaste can lead to cord complications such as the ones assessed in the study, our research showed no significant association between the substance used and cord complications. It is plausible that the very small sample size (6%) of those who used substances other than methylated spirit and chlorhexidine did not allow for a comprehensive comparison. Participants’ recall bias could also be a consideration.

### Limitations

As a limitation, the study was restricted to two facilities and may not represent the whole country. Also, the participants interviewed were caregivers of children aged one week to one year. This poses the challenge of a recall bias. Another limitation is the fact that this is a cross-sectional study. The nature of this study can only establish associations and not causation.

## Conclusions

Only a small fraction of the mothers experienced negative outcomes. Factors such as handwashing, the length of time it took for the cord to fall off, and who was recommending the cord care method had an association with the development of a negative outcome and these were not affected by relevant socio-demographic factors. The substance used in caring for the cord did not have a significant effect on the negative outcomes. We recommend that the Ghana Health Service should intensify education on handwashing before cord care among caregivers. There should be education among the populace on the need to seek advice on health from healthcare workers. This can reduce the risk of cord complications. A randomised control or cohort study should be done to compare the cord outcome using methylated spirit and chlorhexidine.

## Figures and Tables

**Table 1:** Demographic characteristics and cord care practices with cord bleeding

Characteristics	N = 453^1^	No Cord Bleeding n = 440^1^	Cord Bleeding n = 13^1^	p-value^2^
**Facility**				0.560
Ejisu government hospital	279 (61.6)	272 (97.5)	7 (2.5)	
Kumasi south hospital	174 (38.4)	168 (96.5)	6 (3.5)	
**Age of child (days)**				0.204^W^
	94 (60, 180)	95 (60, 180)	74 (40, 134)	
**Mother’s age (years)**				0.294^T^
	29.8 (± 5.3)	29.9 (± 5.3)	28.3 (±5.5)	
**Mother’s education level**				0.455^F^
No formal education	25 (5.5)	23 (92.0)	2 (8.0)	
Primary	32 (7.1)	32 (100.0)	0 (0.0)	
Junior high school	167 (36.9)	162 (97.0)	5 (3.0)	
Senior high school	156 (34.4)	151 (96.8)	5 (3.2)	
Tertiary	73 (16.1)	72 (98.6)	1 (1.4)	
**Occupation**				0.088
Unemployed	90 (19.9)	85 (94.4)	5 (5.6)	
Employed	363 (80.1)	355 (97.8)	8 (2.2)	
**Marital status**				0.492^F^
Single	125 (27.6)	122 (97.6)	3 (2.4)	
Married	313 (69.1)	304 (97.1)	9 (2.9)	
Cohabitation	15 (3.3)	14 (93.3)	1 (6.7)	
**Religion**				0.512^F^
Christian	429 (94.7)	417 (97.2)	12 (2.8)	
Muslim	24 (5.3)	23 (95.8)	1 (4.2)	
**Attended ANC**				1.000^F^
No	2 (0.4)	2 (100.0)	0 (0.0)	
Yes	451 (99.6)	438 (97.1)	13 (2.9)	
**Monthly income (GHC)**				0.027^W^*
	500 (200, 750)	500 (200, 775)	0 (0, 600)	
**Parity**				0.814^W^
	2 (1, 3)	2 (1, 3)	2 (1, 3)	
**Place of delivery**				1.000^F^
Hospital	446 (98.5)	433 (97.1)	13 (2.9)	
Home	7 (1.5)	7 (100.0)	0 (0.0)	
**Mode of delivery**				0.758^F^
Caesarean section	124 (27.4)	120 (96.8)	4 (3.2)	
Vaginal delivery	329 (72.6)	320 (97.3)	9 (2.7)	
**Substance used to clean cord**				1.000^F^
Methylated spirit	407 (89.8)	394 (96.8)	13 (3.2)	
Chlorhexidine	15 (3.3)	15 (100.0)	0 (0.0)	
Nothing	4 (0.9)	4 (100.0)	0 (0.0)	
Others^a^	27 (6.0)	27 (100.0)	0 (0.0)	
**Substance applied to cord after cleaning it**				0.549^F^
Antibiotic	35 (7.7)	33 (94.3)	2 (5.7)	
Herbs	6 (1.3)	6 (100.0)	0 (0.0)	
Nothing	363 (80.2)	353 (97.3)	10 (2.7)	
Others^b^	49 (10.8)	48 (97.9)	1 (2.1)	
**What was used to tie the baby’s cord**				1.000^F^
Clamp	443 (97.8)	430 (97.1)	13 (2.9)	
Don’t know	1 (0.2)	1 (100.0)	0 (0.0)	
Thread	9 (2.0)	9 (100.0)	0 (0.0)	
**Cord exposed after drying**				0.049*
No	133 (29.4)	126 (94.7)	7 (5.3)	
Yes	320 (70.6)	314 (98.1)	6 (1.9)	
**Who recommended the cord care practice**				0.068^F^
Health staff	417 (92.1)	406 (97.4)	11 (2.6)	
Relative	34 (7.5)	33 (97.1)	1 (2.9)	
Others^c^	2 (0.4)	1 (50.0)	1 (50.0)	
**Days the cord took to fell off**				0.547^W^
	5 (4, 7)	5 (4, 7)	5 (4, 7)	
**Washed hands before cord cleaning**				0.057^F^
No	14 (3.1)	12 (85.7)	2 (14.3)	
Yes	439 (96.9)	428 (97.5)	11 (2.5)	
**How often cord care was done**				0.090^F^
Morning and evening	330 (72.8)	323 (97.9)	7 (2.1)	
After every diaper change	27 (6.0)	24 (88.9)	3 (11.1)	
Once a day	8 (1.8)	8 (100.0)	0 (0.0)	
Others^d^	88 (19.4)	85 (96.6)	3 (3.4)	

1 Mean (standard deviation), Median (IQR), Frequency (%)

2 Pearson’s Chi-squared test, ^T^T-test, ^F^ Fisher’s exact test, ^W^ Wilcoxon rank-sum test

a Toothpaste, Herbs, Salt water

b Toothpaste, Shea butter, Cotton, Powder, Clay, Salt water, Chalk

c Neighbour

d Three to six times daily

* p-values < 0.05

**Table 2:** Demographic characteristics and cord care practices with cord granuloma

Characteristics	No Cord Granuloma n = 451^1^	Cord Granuloma n = 2^1^	p-value^2^
**Facility**			0.526^F^
Ejisu government hospital	277 (99.3)	2 (0.7)	
Kumasi south hospital	174 (100.0)	0 (0.0)	
**Age of child (days)**			0.185^W^
	94 (60, 180)	47 (14, 80)	
**Mother’s age (years)**			0.825^T^
	29.8 (± 5.3)	29.0 (± 2.8)	
**Mother’s education level**			0.610^F^
No formal education	25 (100.0)	0 (0.0)	
Primary	32 (100.0)	0 (0.0)	
Junior high school	166 (99.4)	1 (0.6)	
Senior high school	156 (100.0)	0 (0.0)	
Tertiary	72 (98.6)	1 (1.4)	
**Occupation**			1.000^F^
Unemployed	90 (100.0)	0 (0.0)	
Employed	361 (99.5)	2 (0.5)	
**Marital status**			1.000^F^
Single	125 (100.0)	0 (0.0)	
Married	311 (99.4)	2 (0.6)	
Cohabitation	15 (100.0)	0 (0.0)	
**Religion**			1.000^F^
Christian	427 (99.5)	2 (0.5)	
Muslim	24 (100.0)	0 (0.0)	
**Attended ANC**			1.000^F^
No	2 (100.0)	0 (0.0)	
Yes	449 (99.6)	2 (0.4)	
**Monthly income (GHC)**			0.189^W^
	500 (200, 750)	1300 (600, 2000)	
**Parity**			0.901^W^
	2 (1, 3)	3 (1, 4)	
**Place of delivery**			1.000^F^
Hospital	444 (99.5)	2 (0.5)	
Home	7 (100.0)	0 (0.0)	
**Mode of delivery**			0.473^F^
Caesarean section	123 (99.2)	1 (0.8)	
Vaginal delivery	328 (99.7)	1 (0.3)	
**Substance used to clean cord**			1.000^F^
Methylated spirit	405 (99.5)	2 (0.5)	
Chlorhexidine	15 (100.0)	0 (0.0)	
Nothing	4 (100.0)	0 (0.0)	
Others^a^	27 (100.0)	0 (0.0)	
**Substance applied to cord after cleaning it**			1.000^F^
Antibiotic	35 (100.0)	0 (0.0)	
Herbs	6 (100.0)	0 (0.0)	
Nothing	361 (99.5)	2 (0.5)	
Others^b^	49 (100.0)	0 (0.0)	
**What was used to tie the baby’s cord**			1.000^F^
Clamp	441 (99.5)	2 (0.5)	
Don’t know	1 (100.0)	0 (0.0)	
Thread	9 (100.0)	0 (0.0)	
**Cord exposed after drying**			0.501^F^
No	132 (99.3)	1 (0.7)	
Yes	319 (99.7)	1 (0.3)	
**Who recommended the cord care practice**			1.000^F^
Health staff	415 (99.5)	2 (0.5)	
Relative	34 (100.0)	0 (0.0)	
Others^c^	2 (100.0)	0 (0.0)	
**Days the cord took to fell off**			0.312^W^
	5 (4, 7)	13 (5, 21)	
**Washed hands before cord cleaning**			1.000^F^
No	14 (100.0)	0 (0.0)	
Yes	437 (99.5)	2 (0.5)	
**How often cord care was done**			1.000^F^
Morning and evening	328 (99.4)	2 (0.6)	
After every diaper change	27 (100.0)	0 (0.0)	
Once a day	8 (100.0)	0 (0.0)	
Others^d^	88 (100.0)	0 (0.0)	

1 Mean (standard deviation), Median (IQR), Frequency (%)

2 Pearson’s Chi-squared test, ^T^T-test, ^F^ Fisher’s exact test, ^W^ Wilcoxon rank-sum test

a Toothpaste, Herbs, Salt water

b Toothpaste, Shea butter, Cotton, Powder, Clay, Salt water, Chalk

c Neighbour

d Three to six times daily

**Table 3:** Demographic characteristics and cord care practices with cord infection

Characteristics	No Cord Infection n = 437^1^	Cord Infection n = 16^1^	p-value^2^
**Facility**			0.549
Ejisu government hospital	268 (96.1)	11 (3.9)	
Kumasi south hospital	169 (97.1)	5 (2.9)	
**Age of child (days)**			0.204^W^
	91 (60, 180)	119 (77, 270)	
**Mother’s age (years)**			0.557^T^
	29.9 (± 5.3)	29.1 (± 5.4)	
**Mother’s education level**			0.194^F^
No formal education	22 (88.0)	3 (12.0)	
Primary	32 (100.0)	0 (0.0)	
Junior high school	161 (96.4)	6 (3.6)	
Senior high school	152 (97.4)	4 (2.6)	
Tertiary	70 (95.9)	3 (4.1)	
**Occupation**			0.245
Unemployed	85 (94.4)	5 (5.6)	
Employed	352 (97.0)	11 (3.0)	
**Marital status**			0.318^F^
Single	118 (94.4)	7 (5.6)	
Married	304 (97.1)	9 (2.9)	
Cohabitation	15 (100.0)	0 (0.0)	
**Religion**			0.205^F^
Christian	415 (96.7)	14 (3.3)	
Muslim	22 (91.7)	2 (8.3)	
**Attended ANC**			0.069^F^
No	1 (50.0)	1 (50.0)	
Yes	436 (96.7)	15 (3.3)	
**Monthly income (GHC)**			0.138^W^
	500 (200, 750)	300 (0, 600)	
Parity			0.573^W^
	2 (1, 3)	2 (1, 3)	
**Place of delivery**			0.224^F^
Hospital	432 (96.6)	15 (3.4)	
Home	6 (85.7)	1 (14.3)	
**Mode of delivery**			0.776^F^
Caesarean section	119 (96.9)	5 (4.0)	
Vaginal delivery	318 (96.7)	11 (3.3)	
**Substance used to clean cord**			0.126^F^
Methylated spirit	393 (96.6)	14 (3.4)	
Chlorhexidine	14 (93.3)	1 (6.7)	
Nothing	3 (75.0)	1 (25.0)	
Others^a^	27 (100.0)	0 (0.0)	
**Substance applied to cord after cleaning it**			0.908^F^
Antibiotic	34 (97.1)	1 (2.9)	
Herbs	6 (100.0)	0 (0.0)	
Nothing	350 (96.4)	13 (1.6)	
Others^b^	47 (95.9)	2 (4.1)	
**What was used to tie the baby’s cord**			0.305^F^
Clamp	428 (96.6)	15 (3.4)	
Don’t know	1 (100.0)	0 (0.0)	
Thread	8 (88.9)	1 (11.1)	
**Cord exposed after drying**			0.198
No	126 (94.7)	7 (5.3)	
Yes	311 (97.2)	9 (2.8)	
**Who recommended the cord care practice**			0.173^F^
Health staff	404 (96.9)	13 (3.1)	
Relative	31 (91.2)	3 (8.8)	
Others^c^	2 (100.0)	0 (0.0)	
**Days the cord took to fell off**			0.075^W^
	5 (4, 7)	7 (4, 8)	
**Washed hands before cord cleaning**			0.083^F^
No	12 (85.7)	2 (14.3)	
Yes	425 (96.8)	14 (3.2)	
**How often cord care was done**			0.523^F^
Morning and evening	318 (96.4)	12 (3.6)	
After every diaper change	25 (92.6)	2 (7.4)	
Once a day	8 (100.0)	0 (0.0)	
Others^d^	86 (97.7)	2 (2.3)	

1 Mean (standard deviation), Median (IQR), Frequency (%)

2 Pearson’s Chi-squared test, ^T^T-test, ^F^ Fisher’s exact test, ^W^ Wilcoxon rank-sum test

a Toothpaste, Herbs, Salt water

b Toothpaste, Shea butter, Cotton, Powder, Clay, Salt water, Chalk

c Neighbour

d Three to six times daily

**Table 4:** Logistic regression for predictors associated with cord bleeding

	Crude			Adjusted		
Characteristics	OR^1^	95% CI^3^	p-value	OR^2^	95% CI^3^	p-value
**Facility**						
Ejisu^a^	Ref					
Kumasi^b^	1.39	0.46–4.20	0.562			
**Age of child (days)**						
	1.00	0.99–1.00	0.436			
**Mother’s age (years)**						
	0.94	0.85–1.05	0.294			
**Mother’s education level**						
No formal education	Ref					
Primary	-	-	-			
JHS	0.35	0.07–1.94	0.232			
SHS	0.38	0.07–2.08	0.265			
Tertiary	0.16	0.01–1.84	0.142			
**Occupation**						
Unemployed	Ref					
Employed	0.38	0.12–1.20	0.100			
**Marital status**						
Single	Ref					
Married	1.20	0.32–4.52	0.783			
Cohabitation	2.90	0.28–29.85	0.370			
**Religion**						
Christian	Ref					
Muslim	1.51	0.19–12.13	0.698			
**Attended ANC**						
No	Ref					
Yes	-	-	-			
**Monthly income (GHC)**						
	1.00	0.99–1.00	0.148			
**Parity**						
	0.98	0.65–1.49	0.936			
**Place of delivery**						
Hospital	Ref					
Home	-	-	-			
**Mode of delivery**						
Caesarean section	Ref					
Vaginal delivery	0.84	0.26–2.79	0.781			
**Substance used to clean cord**						
Methylated spirit	Ref					
Chlorhexidine	-	-	-			
Nothing	-	-	-			
Others^c^	-	-	-			
**Substance applied to cord after cleaning it**						
Antibiotic	Ref					
Herbs	-	-	-			
Nothing	0.47	0.10–2.22	0.339			
Others^d^	0.34	0.03–3.95	0.391			
**What was used to tie the baby’s cord**						
Clamp	Ref					
Don’t know	-	-	-			
Thread	-	-	-			
**Cord exposed after drying**						
No	Ref					
Yes	0.34	0.11–1.04	0.059			
**Who recommended the cord care practice**						
Health staff	Ref			Ref		
Relative	1.12	0.14–8.93	0.916	ϕ		
Others^e^	36.91	2.17–629.06	0.013*	42	2.45–720.21	0.010*
**Days the cord took to fell off**						
	0.90	0.70–1.15	0.409			
**Washed hands before cord cleaning**						
No	Ref			Ref		
Yes	0.15	0.31–0.77	0.023*	0.13	0.03–0.67	0.015*
**How often cord care was done**						
Morning and evening	Ref					
After every diaper change	5.77	1.40–23.73	0.015*			
Once a day	-	-	-			
Others^f^	1.63	0.41–6.43	0.486			

1 Crude Odds Ratio

2 Adjusted Odds Ratio

3 95% Confidence Interval

^Ref^Reference group

a Ejisu Government Hospital

b Kumasi South Hospital

c Toothpaste, Herbs, Salt water

d Toothpaste, Shea butter, Cotton, Powder, Clay, Salt water, Chalk

e Neighbour

f Three to six times daily

ϕ Factors with p-values > 0.05 were dropped from the analysis

^–^Stata couldn’t compute the estimates due to low outcome observation

* Significant with p-values < 0.05

**Table 5:** Logistic regression for predictors associated with cord granuloma

	Crude			Adjusted		
Characteristics	OR^1^	95% CI^3^	p-value	OR^2^	95% CI^3^	p-value
**Facility**						
Ejisu^a^	Ref					
Kumasi^b^	-	-	-			
**Age of child (days)**						
	0.97	0.92–1.02	0.223			
**Mother’s age (years)**						
	0.97	0.74–1.27	0.824			
**Mother’s education level**						
No formal education	Ref					
Primary	-	-	-			
JHS	0.43	0.03–7.03	0.557			
SHS	-	-	-			
Tertiary	-	-	-			
**Occupation**						
Unemployed	Ref					
Employed	-	-	-			
**Marital status**						
Single	Ref					
Married	-	-	-			
Cohabitation	-	-	-			
**Religion**						
Christian	Ref					
Muslim	-	-	-			
**Attended ANC**						
No	Ref					
Yes	-	-	-			
**Monthly income (GHC)**						
	1.00	1.00–1.00	0.242			
**Parity**						
	1.13	0.44–2.88	0.802			
**Place of delivery**						
Hospital	Ref					
Home	-	-	-			
**Mode of delivery**						
Caesarean section	Ref					
Vaginal delivery	0.38	0.02–6.04	0.489			
**Substance used to clean cord**						
Methylated spirit	Ref					
Chlorhexidine	-	-	-			
Nothing	-	-	-			
Others^c^	-	-	-			
**Substance applied to cord after cleaning it**						
Antibiotic	Ref					
Herbs	-	-	-			
Nothing	-	-	-			
Others^d^	-	-	-			
**What was used to tie the baby’s cord**						
Clamp	Ref					
Don’t know	-	-	-			
Thread	-	-	-			
**Cord exposed after drying**						
No	Ref					
Yes	0.41	0.03–6.66	0.534			
**Who recommended the cord care practice**						
Health staff	Ref					
Relative	-	-	-			
Others^e^	-	-	-			
**Days the cord took to fell off**						
	1.00	0.98–1.03	0.754			
**Washed hands before cord cleaning**						
No	Ref					
Yes	-	-	-			
**How often cord care was done**						
Morning and evening	Ref					
After every diaper change	-	-	-			
Once a day	-	-	-			
Others^f^	-	-	-			

1 Crude Odds Ratio

2 Adjusted Odds Ratio

3 95% Confidence Interval

^Ref^Reference group

a Ejisu Government Hospital

b Kumasi South Hospital

c Toothpaste, Herbs, Salt water

d Toothpaste, Shea butter, Cotton, Powder, Clay, Salt water, Chalk

e Neighbour

f Three to six times daily

ϕ Factors with p-values > 0.05 were dropped from the analysis

^–^Stata couldn’t compute the estimates due to low outcome observation

* Significant with p-values < 0.05

**Table 6:** Logistic regression for predictors associated with cord infection

	Crude			Adjusted		
Characteristics	OR^1^	95% CI^3^	p-value	OR^2^	95% CI^3^	p-value
**Facility**						
Ejisu^a^	Ref					
Kumasi^b^	0.72	0.25–2.11	0.550			
**Age of child (days)**						
	1.00	1.00–1.01	0.122			
**Mother’s age (years)**						
	0.97	0.88–1.07	0.556			
**Mother’s education level**						
No formal education	Ref					
Primary	-	-	-			
JHS	0.27	0.06–1.17	0.081			
SHS	0.19	0.04–0.92	0.039*			
Tertiary	0.31	0.06–1.67	0.174			
**Occupation**						
Unemployed	Ref					
Employed	0.53	0.18–1.57	0.252			
**Marital status**						
Single	Ref					
Married	0.50	0.18–1.37	0.178			
Cohabitation	-	-	-			
**Religion**						
Christian	Ref					
Muslim	2.69	0.58–12.60	0.208			
**Attended ANC**						
No	Ref			Ref		
Yes	0.03	0.00–0.58	0.019*	0.03	0.00–0.56	0.018*
**Monthly income (GHC)**						
	1.00	1.00–1.00	0.342			
**Parity**						
	0.88	0.59–1.33	0.551			
**Place of delivery**						
Hospital	Ref					
Home	4.79	0.54–42.31	0.159			
**Mode of delivery**						
Caesarean section	Ref					
Vaginal delivery	0.82	0.28–2.42	0.724			
**Substance used to clean cord**						
Methylated spirit	Ref					
Chlorhexidine	2.01	0.25–16.34	0.516			
Nothing	9.36	0.91–95.70	0.059			
Others^c^	-	-	-			
**Substance applied to cord after cleaning it**						
Antibiotic	Ref					
Herbs	-	-	-			
Nothing	1.26	0.16–9.95	0.825			
Others^d^	1.45	0.13–16.61	0.767			
**What was used to tie the baby’s cord**						
Clamp	Ref					
Don’t know	-	-	-			
Thread	3.57	0.42–30.37	0.245			
**Cord exposed after drying**						
No	Ref					
Yes	0.52	0.19–1.43	0.205			
**Who recommended the cord care practice**						
Health staff	Ref					
Relative	3.01	0.81–11.12	0.099			
Others^e^	-	-	-			
**Days the cord took to fell off**						
	1.00	0.98–1.02	0.989			
**Washed hands before cord cleaning**						
No	Ref			Ref		
Yes	0.20	0.04–0.97	0.045*	0.20	0.04–0.98	0.047*
**How often cord care was done**						
Morning and evening	Ref					
After every diaper change	2.12	0.45–10.00	0.342			
Once a day	-	-	-			
Others^f^	0.62	0.14–2.81	0.531			

1 Crude Odds Ratio

2 Adjusted Odds Ratio

3 95% Confidence Interval

^Ref^Reference group

a Ejisu Government Hospital

b Kumasi South Hospital

c Toothpaste, Herbs, Salt water

d Toothpaste, Shea butter, Cotton, Powder, Clay, Salt water, Chalk

e Neighbour

f Three to six times daily

ϕ Factors with p-values > 0.05 were dropped from the analysis

^–^Stata couldn’t compute the estimates due to low outcome observation

* Significant with p-values < 0.05

## Data Availability

The anonymized datasets generated and/ or analyzed during the current study are not publicly available but are available from the corresponding author on reasonable request.

## Abbreviations

CHRPE: Committee on Human Research, Publications, and Ethics
CI: Confidence Interval
IQR: Interquartile Range
KNUST: Kwame Nkrumah University of Science and Technology
NMR: Neonatal Mortality Rate
OR: Odds Ratio
SSA: Sub-Saharan Africa
WHO: World Health Organization
